# CHOP aggravates hepatocyte apoptosis upon endoplasmic reticulum stress by downregulating autophagy

**DOI:** 10.1016/j.cstres.2025.02.005

**Published:** 2025-02-27

**Authors:** Jia-Yu Wu, Bing Han, Ting Yang, Lu Zheng, Yi-Xin Guo, Jia-Yao Li, Xiao-Yu Guo, Huan-Huan Yin, Ru-Jia Xie

**Affiliations:** 1 Department of Pathophysiology, College of Basic Medical Sciences, Guizhou Medical University, Guiyang, Guizhou Province, China; 2 Guizhou Provincial Key Laboratory of Pathogenesis and Drug Research on Common Chronic Diseases, College of Basic Medical Sciences, Guizhou Medical University, Guiyang, Guizhou Province, China

**Keywords:** C/EBP homologous protein, Autophagy, Endoplasmic reticulum stress, Apoptosis, Hepatocyte

## Abstract

Endoplasmic reticulum (ER) stress-induced apoptosis plays a crucial role in various liver diseases. Hepatocytes respond to ER stress by activating the unfolded protein response and autophagy, which is essential for maintaining ER homeostasis. However, failure to restore ER balance via autophagy contributes to apoptosis. In this study, we aimed to explore the role of C/EBP homologous protein (CHOP) in regulating ER stress-induced apoptosis in rat hepatocytes. We found that CHOP downregulates autophagy, aggravating apoptosis. Our results revealed that inhibition of CHOP expression enhanced autophagy and reduced DTT-induced apoptosis in BRL-3A cells, whereas CHOP overexpression worsened apoptosis. Chromatin immunoprecipitation assays revealed that CHOP negatively regulates autophagy-related genes, such as ATG12, ATG5, and LC3. These findings suggest that CHOP modulation plays a crucial role in ER stress-induced hepatocyte apoptosis by regulating autophagy.

## Highlights

• Endoplasmic reticulum (ER) stress triggers apoptosis in liver diseases *via* C/EBP homologous protein (CHOP) and autophagy.
• Autophagy is crucial for maintaining ER homeostasis in hepatocytes.
• CHOP overexpression leads to reduced autophagy and increased apoptosis.
• Pretreatment with rapamycin mitigates ER stress-related cell death.
• Silencing CHOP enhances autophagy and decreases apoptosis in stressed cells.

## Introduction

The prominence of hepatocyte apoptosis in the process of liver diseases has been widely concerning.^1^, ^2^, ^3^ A series of investigations unveiled that endoplasmic reticulum (ER) stress-associated hepatocyte apoptosis is the core event in the process of various hepatic impairments.^4^, ^5^, ^6^, ^7^, ^8^ Multiple potentially harmful stimuli, such as various pathological conditions and pharmacological insults, can perturb the normal ER function of hepatocytes and lead to ER stress. The hepatocytes react to stressors by initiating unfolded protein response (UPR) and enhancing autophagy, which helps alleviate the impairments of ER and reestablish its homeostasis.^9^, ^10^, ^11^ The UPR is a cellular homeostatic signaling pathway, which halts the translation of general protein and enhances the gene transcription of ER-localized chaperones.^12^, ^13^ Autophagy is a pivotal procedure for sustaining normal ER function, through degradation of damaged ER fragments and removal of aberrant protein aggregates in the ER lumen.^14^, ^15^ Recent findings indicate that a failure to restore ER homeostasis *via* autophagy is harmful to hepatocytes and contributes to ER stress-associated apoptosis; however, the potential regulatory mechanisms need further validation.^16^

C/EBP homologous protein (CHOP), a transcription factor, that drives the transcription of proapoptotic genes. Some scholars have revealed that CHOP can initiate the apoptotic response by activating proapoptotic proteins, such as Bim and death receptor, and reducing antiapoptotic proteins, such as Bcl-2.^17^ Recently, investigations have indicated that CHOP can promote ER stress-related apoptosis by downregulating autophagy, but the underlying precise mechanism is unknown.^18^ Our previous study unveiled that CHOP expression was upregulated and apoptosis was significantly augmented during dithiothreitol (DTT)-induced ER stress in hepatocytes. However, it is unclear whether CHOP can aggravate hepatocyte apoptosis upon ER stress by downregulating autophagy. Our experiment was conducted to reveal the role of CHOP in ER stress-associated cellular apoptosis; it might offer a new guide for therapy in liver diseases.

## Results

### Impact of rapamycin or 3-methyladenine pretreatment on the proliferation of BRL-3A cells undergoing ER stress

**Fig. 1:**
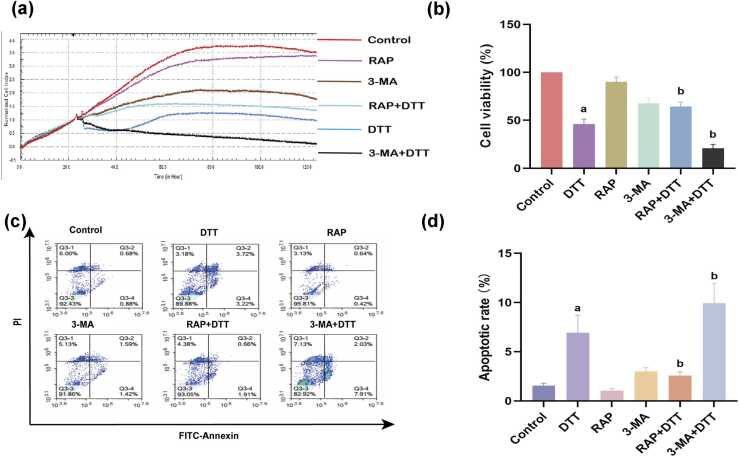
Effects of RAP and 3-MA pretreatment on BRL-3A cell proliferation and apoptosis under ER stress. BRL-3A cells were individually treated with DTT, RAP, or 3-MA. RAP or 3-MA pretreatment was performed on BRL-3A cells, and followed by DTT treatment. (a) and (b) Dynamic monitoring of the normalized cell index of BRL-3A cells by real-time cellular analysis xCELLi-gene system. (c) and (d) Apoptotic cell proportions were assessed using flow cytometry. Data are shown as mean ± standard deviation. ^a^*P* < 0.05 *versus* control group, ^b^*P* < 0.05 *versus* DTT group (n = 3). Abbreviations used: 3-MA, 3-methyladenine; DTT, dithiothreitol; ER, endoplasmic reticulum; PI, propidium iodide; RAP, rapamycin.

Earlier, studies found that treatment with DTT could result in a notable reduction in the proliferation of BRL-3A cells, and the inhibitory effect of DTT was dose-dependent. 3-Methyladenine (3-MA) pretreatment, followed by DTT treatment for 24 h, inhibited cell proliferation in BRL-3A cells. However, rapamycin (RAP) pretreatment did not (Figure 1(a) and (b)).

### Impact of RAP or 3-MA pretreatment on apoptosis of BRL-3A cells undergoing ER stress

Persistent ER stress could initiate the ER stress-associated apoptosis signaling pathway. Treatment with DTT was found to significantly increase BRL-3A cell apoptosis in our previous study. In the present study, we observed that RAP pretreatment in BRL-3A cells markedly decreased DTT-induced apoptosis. However, 3-MA pretreatment accelerated DTT-induced apoptosis (Figure 1(c) and (d)).

### Impact of RAP or 3-MA pretreatment on the level of ER stress-related molecules in BRL-3A cells undergoing ER stress

**Fig. 2:**
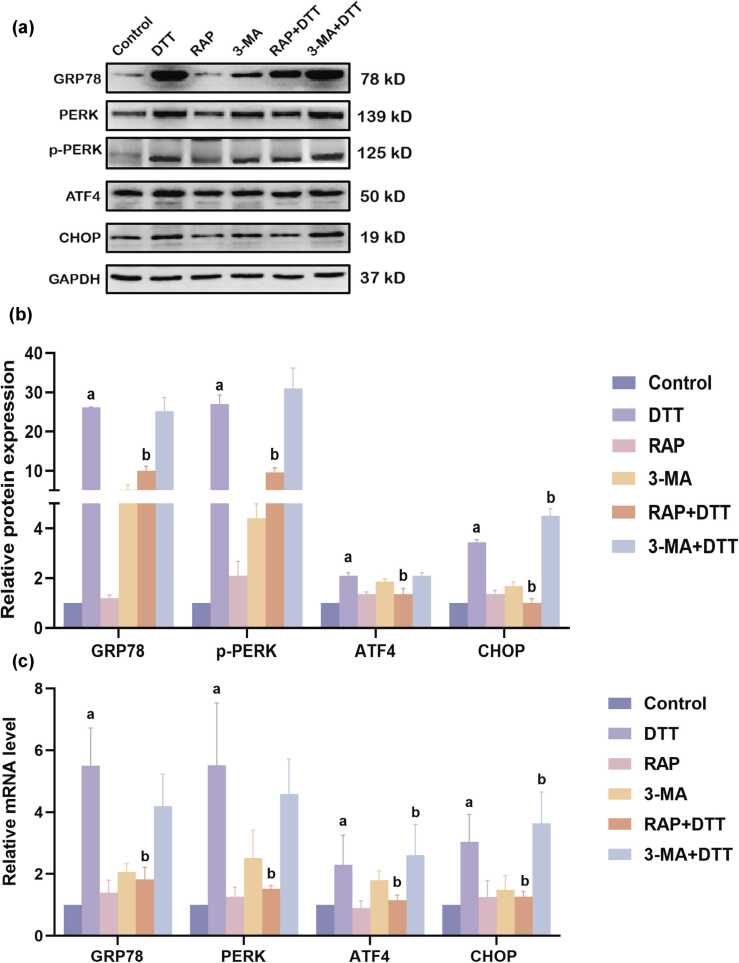
Effects of RAP or 3-MA pretreatment on the expression of ER stress-related molecules in BRL-3A cells undergoing ER stress*.* BRL-3A cells were individually treated with DTT, RAP, or 3-MA. Then, RAP or 3-MA pretreatment was performed on BRL-3A cells followed by DTT treatment. (a) and (b) Western blotting was used to assess proteins involved in the ER stress pathway, with relative expression levels normalized to β-actin and further standardized to the control group. (c) The mRNA level of ER stress-related gene was detected using PCR, with data normalized to the control group. Data are shown as mean ± standard deviation. ^a^*P* < 0.05 *versus* control group, ^b^*P* < 0.05 *versus* DTT group (n = 3). Abbreviations used: 3-MA, 3-methyladenine; DTT, dithiothreitol; ER, endoplasmic reticulum; RAP, rapamycin.

Previous research indicated elevated levels of ER stress markers, such as glucose-regulated protein 78 (GRP78), in BRL-3A cells treated with DTT. This was accompanied by increased phosphorylation of PRKR-like endoplasmic reticulum kinase (PERK), as well as higher levels of activating transcription factor 4 (ATF4) and CHOP. In our current study, pretreatment with RAP significantly decreased the levels of GRP78, p-PERK, ATF4, and CHOP. Conversely, pretreatment with 3-MA led to a notable increase in ATF4 and CHOP expression in DTT-treated BRL-3A cells, though it did not affect GRP78 or p-PERK levels (Figure 2(a)-(c)).

### The promotion effect of CHOP on DTT-mediated hepatocyte apoptosis

**Fig. 3:**
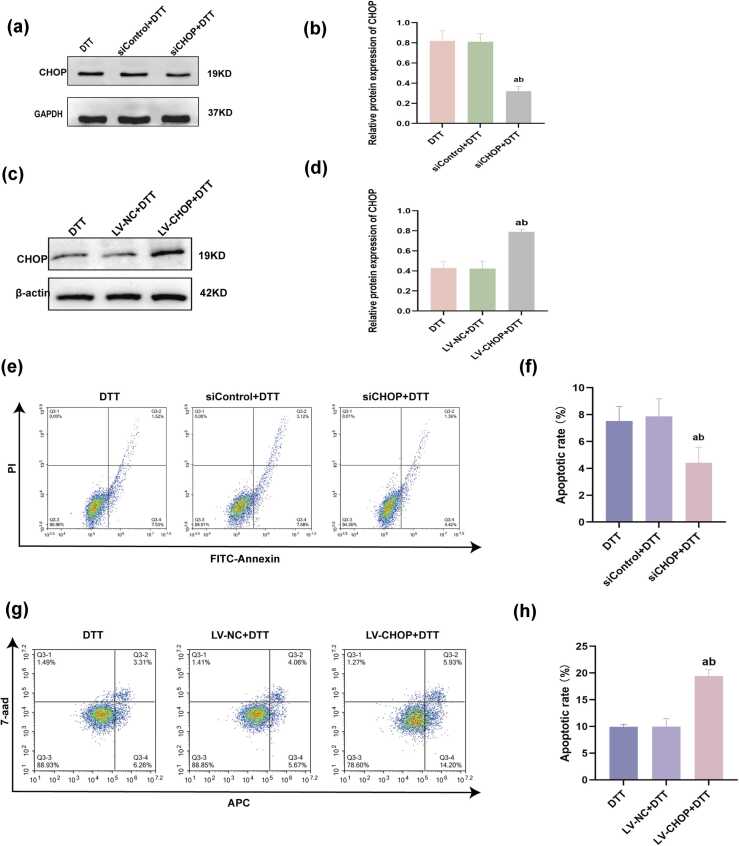
Impact of CHOP silencing and overexpression on apoptosis in BRL-3A cells subjected to ER stress. BRL-3A cells were either transfected with CHOP siRNAs or infected with CHOP lentivirus, followed by 24 h of DTT treatment. (a-d) Western blot was used to detect the transfection efficiency of siRNA and lentivirus. (e-h) Apoptotic cell proportions were analyzed using a flow cytometer. Data are shown as mean ± standard deviation. ^a^*P* < 0.05 *versus* DTT group, ^b^*P* < 0.05 *versus* sicontrol group (n = 3). Abbreviations used: CHOP, C/EBP homologous protein; DTT, dithiothreitol; ER, endoplasmic reticulum; PI, propidium iodide.

To examine the impact of CHOP on DTT-mediated apoptosis, the lentiviral vector system was applied to established BRL-3A cells with CHOP overexpression, and CHOP siRNA transfection was used to decrease the expression of CHOP. After infection, 2.0 mmol/L DTT was used to treat cells for 24 h. Overexpression and knockdown efficiency were detected *via* western blot analysis (Figure 3(a) and (c)). The results indicated that BRL-3A cells had a reduced apoptotic rate after transfection with CHOP knockdown (Figure 3(e)). The apoptotic rate increased with CHOP overexpression (Figure 3(g)), suggesting that CHOP promotes DTT-mediated apoptosis in BRL-3A cells.

### CHOP inhibits the autophagic level and directly interacts with ATG12, ATG5, and LC3 in BRL-3A cells undergoing ER stress

**Fig. 4:**
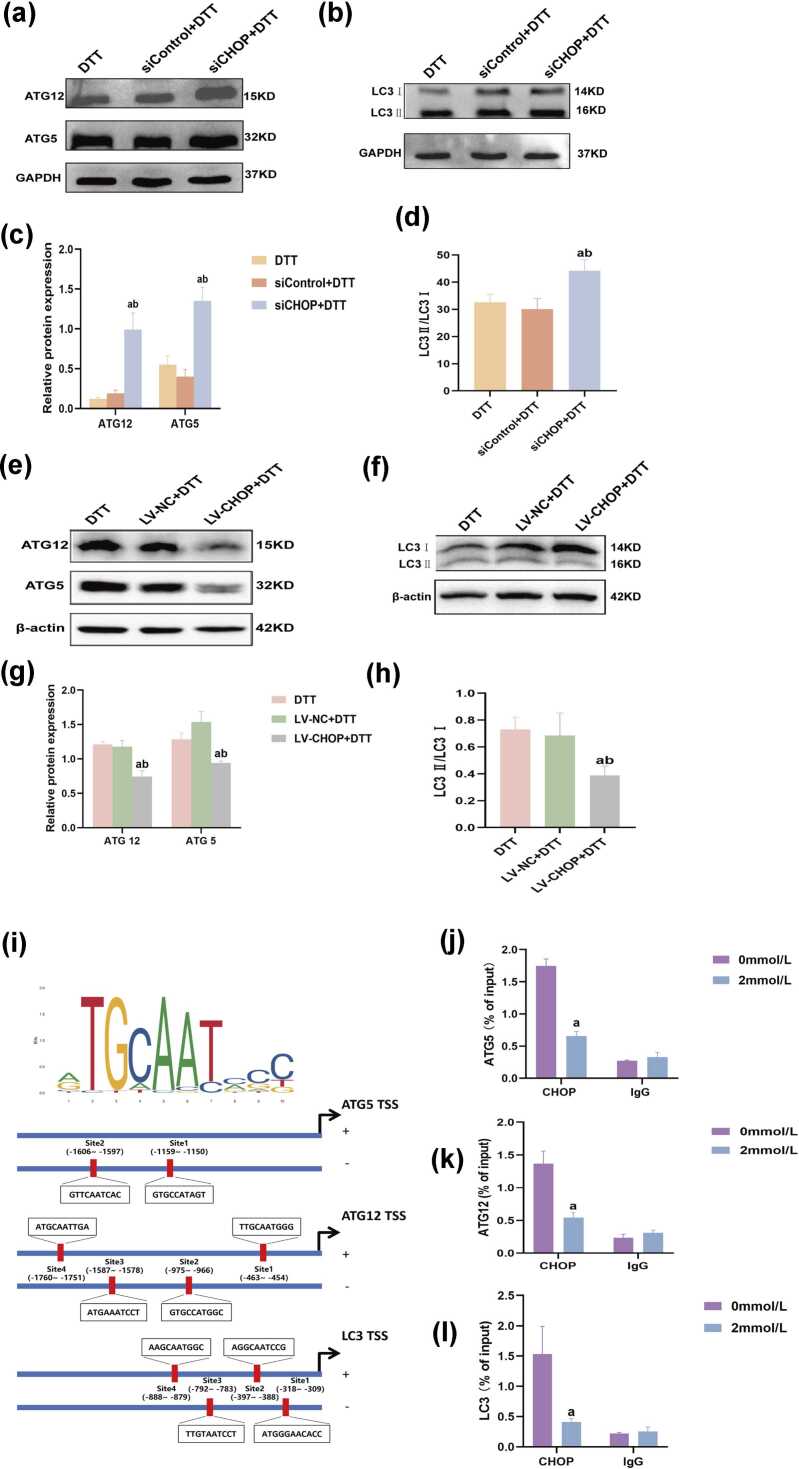
Effects of CHOP silencing and overexpression on the expression of autophagy-related proteins in BRL-3A cells undergoing ER stress*.* (a and b) BRL-3A cells were transfected with CHOP small interfering RNAs and then treated with DTT for 24 h. (e and f) BRL-3A cells were infected with CHOP lentivirus and then treated with DTT for 24 h. (a-h) Western blot was used to detect the expression of autophagy-related proteins, and relative protein expression levels were normalized to β-actin. (i) Predicted CHOP binding sites within the promoter regions of ATG12, ATG5, and LC3 (spanning 2 kb upstream of the transcription start site) identified using the JASPAR database. (j-l) BRL-3A cells were exposed to 0 or 2 mmol/L DTT for 24 h. ChIP was used to determine the binding of CHOP on ATG5, ATG12, and LC3 promoter. Data are shown as mean ± standard deviation. ^a^*P* < 0.05 *versus* DTT group, ^b^*P* < 0.05 *versus* sicontrol group (n = 3). Abbreviations used: CHOP, C/EBP homologous protein; DTT, dithiothreitol; ER, endoplasmic reticulum.

We examined whether CHOP knockdown or overexpression affected autophagy induced by DTT. DTT-treated BRL-3A cells showed increased expression of autophagy-related molecules, following siRNA-mediated silencing of CHOP. In contrast, opposite trends were detected in CHOP overexpression cells (Figure 4(a)-(h)). These molecules included ATG5, ATG12, and LC3. Specifically, the LC3-I to LC3-II conversion ratio was higher in the CHOP siRNA group than in controls, while it was lower in the LV-CHOP group relative to that in the control and LV-NC groups (Figure 4(b) and (f)). Additionally, chromatin immunoprecipitation (ChIP) assays confirmed that CHOP negatively regulates the transcription of autophagy-related genes such as ATG12, ATG5, and LC3 during ER stress. Predicted CHOP binding sites in the promoter regions of ATG5, ATG12, and LC3 were validated using ChIP-qPCR. The analysis confirmed CHOP binding at site 2 within the ATG5 promoter, site 4 within the ATG12 promoter, and site 2 within the LC3 promoter. These results are consistent with predictions and indicate specific interactions of CHOP with these promoter regions during ER stress. The analysis revealed reduced input percentage (%Input) values for CHOP enrichment at the promoters of ATG12, ATG5, and LC3 in cells treated with DTT compared to the 0 mmol/L group. RT-qPCR results confirmed that amplification was noticeably reduced in the 2 mmol/L DTT group compared to the 0 mmol/L group, suggesting that DTT treatment decreases CHOP binding to the promoter regions of autophagy-related genes during ER stress, potentially reflecting altered transcriptional regulation under these conditions (Figure 4(i)-(l)). All results suggested that CHOP inhibited DTT-induced autophagy in BRL-3A cells.

## Discussion

The ER is a critical modulator of hepatocyte apoptosis.^19^, ^20^, ^21^, ^22^ As a prominent organelle in hepatocytes, the ER contributes to lipid synthesis, calcium storage, and protein folding.^23^, ^24^ When its function is perturbed by various pathological processes or pharmacological agents, including tunicamycin, thapsigargin, and DTT, ER stress occurs.^25^, ^26^ Our previous data unveiled that DTT upregulated ER stress-associated proteins, including ER molecular chaperone, GRP78, ATF4, and CHOP. The hepatocytes initiate an adaptive response upon ER stress, UPR. This adaptive response maintains ER homeostasis by fostering the gene transcription of chaperons and promoting the degradation of aberrant protein aggregates in the ER lumen.^27^, ^28^ A series of studies underscored that the autophagy signaling pathway is activated in hepatocytes reacting to ER stress, which results in the elimination of unfolded/misfolded proteins and plays a pivotal role in reestablishing normal ER function.^29^, ^30^ The present study investigated whether the alteration of autophagy influences ER stress-mediated hepatocyte apoptosis. We found that enhancing autophagy with RAP pretreatment mitigated DTT-induced hepatocyte apoptosis; however, suppression of autophagy with 3-MA pretreatment aggravated DTT-induced hepatocyte apoptosis. The above data indicated that autophagy could rescue hepatocytes from ER stress-mediated cellular injury. Moreover, it was observed that the level of ER stress-related proapoptotic molecules, including ATF4 and CHOP, was notably decreased after RAP pretreatment, suggesting that the enhancement of cellular autophagy could mitigate ER stress-mediated apoptosis. On the contrary, the levels of ATF4 and CHOP were markedly increased after 3-MA pretreatment, suggesting that inhibition of cellular autophagy may enhance the level of the proapoptotic molecules in the ER stress-associated apoptotic pathway and foster cell apoptosis.

CHOP is a prominent transcription factor, which acts as a mediator of apoptosis in ER stress. Some scholars have revealed that CHOP can initiate the apoptotic response by activating proapoptotic proteins, such as Bim and death receptor, and reducing antiapoptotic proteins, such as Bcl-2.^31^, ^32^ More recently, investigations have indicated that CHOP can aggravate ER stress-related apoptosis by regulating autophagy. Numerous studies validated that CHOP was dramatically upregulated in cells exposed to severe stress.^33^, ^34^ Our present data also unveiled that the level of CHOP was dramatically upregulated in BRL-3A cells exposed to DTT, indicating that CHOP might favor ER stress-initiated apoptosis in BRL-3A cells. To determine whether CHOP regulates cellular apoptosis through modulation of the autophagic process, siCHOP transfection, and LV-CHOP infection were done to achieve silencing and overexpression of CHOP in hepatocytes, respectively. To investigate the targeting relationship between CHOP and autophagy-related genes, we conducted a ChIP experiment to detect CHOP in the ATG12, ATG5, and LC3 promoter regions. The results suggested that CHOP could bind directly to ATG12, ATG5, and LC3, and inhibit their transcription in BRL-3A cells exposed to DTT. This finding is consistent with a previous study, which demonstrated that CHOP directly binds to the promoter regions of autophagy-related genes and regulates their transcription in response to stress signals.^34^ Our data showed that CHOP silencing not only notably ameliorated the DTT-triggered hepatocyte apoptosis but also enhanced the level of autophagy, and an opposite trend was shown in CHOP overexpression.

## Conclusion

The failure to restore ER homeostasis *via* autophagy contributes to ER stress-associated apoptosis. Enhancing autophagy with RAP pretreatment mitigated DTT-initiated hepatocyte apoptosis; however, suppression of autophagy with 3-MA pretreatment aggravated DTT-induced hepatocyte apoptosis. The upregulation of CHOP in ER stress procedures could inhibit autophagy and promote hepatocyte apoptosis. In contrast, knockdown of the CHOP gene could attenuate DTT-induced hepatocyte apoptosis.

## Materials and methods

### Cell culture

A cell line from rat BRL-3A (KCB92013YJ) was purchased from the Kunming Cell Bank of the Chinese Academy of Sciences (Kunming, China). An incubation medium supplemented with 1% penicillin-streptomycin (BioInd, Beit-Haemek, Israel) plus 10% fetal bovine serum (Gibco, Grand Island, NY, USA) was used to incubate the cells. Incubation was performed at 37 °C in a 5% carbon dioxide supplemented atmosphere with the medium exchanged every 3 days after three phosphate buffer saline rinses. 2.0 mM DTT (Genview, CA, USA) was utilized to treat cells for 24 h as an ER stress inducer.

### Cell viability assay

The 1 × 10^4^ BRL-3A cells were seeded onto an E-Plate and treated with 2.0 mM DTT, 0.4 μM RAP, and 10 mM 3-MA, respectively. Then, 0.4 μM RAP or 10 mM 3-MA pretreatment was carried out on the BRL-3A cells, and followed by 2.0 mM DTT treatment. The proliferation of BRL-3A cells was continuously monitored using real-time cellular analysis, which can monitor the proliferation of hepatocytes with a label-free assay. Data are presented as mean percentages of vehicle-treated cell proliferation ± SD.

### Flow cytometric analysis

The 5 × 10^5^ BRL-3A cells were added to a 25 mm^2^ culture flask and treated with 2.0 mM DTT, 0.4 μM RAP, and 10 mM 3-MA for 24 h, respectively. Subsequently, 0.4 μM RAP or 10 mM 3-MA pretreatment was performed on BRL-3A cells for 1 h, followed by 2.0 mM DTT treatment for 24 h. Then, cells were harvested and rinsed twice with 10 mL phosphate buffer saline. Following centrifugation for 5 min at 1000 r, the pellets were resuspended in 195 μL of Annexin V-FITC binding buffer. Subsequently, 5 μL of Annexin V-FITC was added, followed by 10 μL of propidium iodide solution (Beyotime, Nanjing, China). Flow cytometry analyses were conducted following a 20-min incubation period at ambient temperature.

### Western blotting analysis

The 1 × 10^6^ BRL-3A cells were subjected to treatment with 2.0 mM DTT, 0.4 μM RAP, and 10 mM 3-MA for 24 h, respectively. Subsequently, 1 h pretreatment with 0.4 μM RAP or 10 mM 3-MA was performed on BRL-3A cells, followed by 2.0 mM DTT treatment for 24 h. Incubated on ice for 10 min, BRL-3A cells were lysed and centrifuged at 12,000 r for 15 min, after which the supernatants containing protein were stored at −40 °C. Equal quantities of protein were separated using an 8–12% SDS-PAGE and subsequently transferred onto PVDF membranes (Merck Millipore, MA, USA). Following blocking with 5% skim milk at room temperature for 2 h, the membranes were incubated overnight at 4 °C with the following primary antibodies: β-actin (1:1000), GRP78 (1:1000), PERK (1:1500), p-PERK (1:1000), ATF4 (1:1500), CHOP (1:1000), LC3 antibody (1:1000), ATG5 antibody (1:1000), and ATG12 antibody (1:500). Following the washing procedure with TBST buffer, the membranes were subjected to a 1 h incubation at room temperature with the corresponding secondary antibodies. Subsequently, visualization was carried out using an ECL reagent (Merck Millipore, MA, USA). Bio-Rad imaging system (Bio-Rad, USA) was used to export protein band intensity.

### RT-qPCR

TRIzol Reagent (Takara, Tokyo, Japan) was used to isolate total RNA from 1 × 10^6^ BRL-3A cells. RNA was converted into complementary DNA templates using the Evo M-MLV RT Kit (AG11705, Accurate Biotechnology, Hunan, China). Gene amplification was conducted using SYBR Premix Ex Taq™ (Takara, Tokyo, Japan). The levels of mRNA expression were standardized to β-actin, and the quantitative primers were produced by Sangon (Shanghai, China).

### CHOP siRNA transfection

The BRL-3A cells, totaling 5 × 10^5^, were placed in a 25 mm^2^ culture flask with DMEM, supplemented with 10% FBS without antibiotics, and incubated at 37 ℃ in a humidified atmosphere with 5% carbon dioxide. Before transfection, the medium was changed routinely without an antibiotic. The cells were divided into three groups at random as follows: normal control, control siRNA, and CHOP siRNA. The siRNA transfection reagent was obtained from Santa Cruz Biotechnology (CA, USA). CHOP protein downregulation was confirmed by western blotting. After transfection, BRL-3A cells were subjected to a 2.0 mM DTT treatment for 24 h. Subsequently, we observed the apoptosis of BRL-3A cells, as well as the expression of CHOP and autophagy-related molecules in hepatocytes.

### Lentivirus infection

The lentiviruses LV-GFP-CHOP and LV-GFP-NC purchased from GENE (Shanghai, China) were applied for CHOP overexpression. At 72 h postinfection, cells were cultured in medium with puromycin (4 μg/mL). The cells were divided into three groups: control, LV-NC, and LV-CHOP. After infection, BRL-3A cells were subjected to a 2.0 mM DTT treatment for 24 h. Afterward, the overexpression efficiency was detected *via* western blot analysis.

### Chromatin immunoprecipitation

ChIP was performed following the ChIP Assay Kits protocol, in accordance with the manufacturer’s instructions (Thermo Fisher Scientific, Waltham, MA, USA). BRL-3A cells were exposed to 0–2 mmol/L DTT for 24 h, then cross-linked with 1% formaldehyde for 10 min at room temperature. The reaction was terminated with 125 mmol/L glycine. After sonication and centrifugation (9000g/min), chromatin fragments of 200–1000 bp were collected. The chromatin was incubated overnight at 4 °C with anti-CHOP (1:50) and rabbit IgG (1:50), followed by DNA purification. DNA from nuclear extracts without antibody incubation served as the input control, and quantitative PCR was used to analyze the DNA from each sample. The %Input was calculated as follows:

%Input=2^Ct(Adjusted Input)−Ct(ChIP)^ ×100,

and the Ct (Adjusted Input) was determined using the formula:

Ct(Adjusted Input)=Ct(Input)−log_2_(DF),

where DF is the dilution factor (10).

### Statistical analysis

The analysis was conducted using SPSS 13.0 statistical software, and the data were presented as mean ± standard deviation. GraphPad Prism software was used to prepare experimental graphs and the one-way ANOVA method was employed for the multivariate comparison. There is statistical significance in differentiation when *P* < 0.05.

### CRediT authorship contribution statement

**Bing Han:** Writing – original draft, Methodology, Investigation. **Jia-Yu Wu:** Writing – original draft, Methodology, Investigation. **Ru-Jia Xie:** Writing – review & editing, Conceptualization. **Huan-Huan Yin:** Formal analysis, Data curation. **Xiao-Yu Guo:** Formal analysis, Data curation. **Jia-Yao Li:** Formal analysis, Data curation. **Yi-Xin Guo:** Formal analysis, Data curation. **Lu Zheng:** Writing – original draft, Methodology, Investigation. **Ting Yang:** Writing – review & editing, Conceptualization.

## Funding and support

The National Natural Science Foundation of China (Grant No. 82260127), Guizhou Provincial Science and Technology Projects (Grant No. Qiankehe Jichu-ZK[2021]365), National Natural Science Foundation Cultivation Project of Guizhou Medical University (Grant No. 20NSP016), and Guizhou Provincial Basic Research Program (Natural Science, No. ZK[2025]542).

## Author contributions

RJX and TY designed the study and revised the manuscript. JYW, BH, and LZ carried out the experiments. JYL, YXG, XYG, and HHY assembled and analyzed the data. The article was written by JYW and RJX. All authors approved the final version of the manuscript.

## Declarations of interest

The authors declare the following financial interests/personal relationships, which may be considered as potential competing interests: Reports a relationship with that includes: Has a patent pending too. If there are other authors, they declare that they have no known competing financial interests or personal relationships that could have appeared to influence the work reported in this paper.

## Data Availability

Data will be made available on request.
